# Improving Health Outcomes and Serving Wider Society: The Potential Role of Understanding and Cultivating Prosocial Purpose Within Health Psychology Research and Practice to Address Climate Change and Social Isolation and Loneliness

**DOI:** 10.3389/fpsyg.2019.01787

**Published:** 2019-08-07

**Authors:** Kiran Kaur Bains, Triece Turnbull

**Affiliations:** ^1^Health Services Research, School of Health Sciences, City, University of London, London, United Kingdom; ^2^Centre for Psychology Ltd., Surrey, United Kingdom; ^3^Department of Health, Psychology and Communities, Manchester Metropolitan University, Manchester, United Kingdom

**Keywords:** purpose, physical health, mental health, protective factors, human development, climate change, social issues

## Abstract

Human beings face unprecedented social and environmental challenges which require collective action and changes in health-related behavior. The threat of climate change is becoming an increasingly urgent issue for humanity and the natural environment. Alongside this, there is evidence that loneliness and social isolation can significantly impact cardiovascular health and mortality through direct and indirect processes, for example by increasing risky behaviors. However, one construct that has so far received little attention in health psychology is that of purpose. Purpose is thought to be self-regulatory; it derives from a greater sense of meaning yet is goal-directed and involves a stable and generalized intention toward accomplishment. The development of a sense of purpose is associated with improved mental and physical health. However, it is possible that one facet of purpose, prosocial orientation, may have a particularly beneficial effect on psychological well-being, increasing generativity and personal growth. Prosocial purpose may also help explain the growth in the number of people in the West who are reducing their meat and dairy intake, which may help mitigate climate change. It may also help explain the rise of civic engagement in environmental volunteering and support for conservation amongst some individuals and communities, which can also confer additional health benefits. Cultivating prosocial purpose may aid engagement in behavior change initiatives which may improve individual health and help address these wider social challenges, such as changing one’s diet to help address climate change, volunteering and engaging in physical activity outdoors to support the environment, and supporting active engagement with vulnerable groups at risk of social isolation and loneliness. Cultivating prosocial purpose may also support self-advocacy for social changes which can benefit community health. It may be possible to cultivate prosocial purpose through interventions which involve experiential and abstract learning experiences that increase empathy, stimulate reflection and lead to meaning-making processes. This may then facilitate development of a sense of prosocial purpose because meaning-making is thought to be a precursor to purpose development. Doing so may be important to engage populations in efforts to combat climate change and address social isolation and loneliness.

## Introduction

Humans face serious threats to their health and well-being due to a variety of issues, including climate change and increased social isolation and loneliness. The latest, widely publicized Intergovenmental Panel on Climate Change (IPCC, 2018) report suggests wide-ranging social changes are required by 2030 to limit temperature rises to 1.5°C in order to manage impacts on human civilization and the natural environment. These include loss of biodiversity, increases in ocean temperature and acidity, and threats to food security, water supplies, and political and social stability (IPCC, 2018; Beylot et al., 2019).

Climate change is already contributing to an increase in a number of negative effects: extreme weather events, which may cause severe injury to individuals; drought and flooding, which can compromise sanitation systems and create greater risk of infectious disease; heat-related illnesses; and respiratory diseases (Barrett et al., 2015; Brittany et al., 2018). These are serious problems that health professionals, including health psychologists, need to better understand to help mitigate climate change and its effects on humanity (Brittany et al., 2018). It is likely that these effects will worsen in the future. In order to mitigate the effects of climate change on psychosocial health, it is important to understand and anticipate them, and develop strategies to combat them, which may include participation in climate-health governance activities (Adams, 2014; Brittany et al., 2018).

Social isolation and loneliness are also key issues facing communities. Social isolation occurs when individuals have few social contacts and limited social networks, whilst loneliness is a subjective feeling of isolation and lack of social connection with wider networks (Newall and Menec, 2019). A person can have few social connections (i.e., be assessed as quite socially isolated) and report little loneliness, but also have many social contacts but report great loneliness (Newall and Menec, 2019).

Previous research suggests that both social isolation and loneliness increase mortality (Leigh-Hunt et al., 2017). Leigh-Hunt et al. (2017) conducted a systematic overview of forty prior systematic reviews and found that high levels of social isolation were associated with an odds ratio [OR] of increased all-cause mortality (excluding suicide) of 1.29 (95% confidence interval [CI] of 1.06–1.56), loneliness with an OR of 1.26 (CI 1.04–1.53), and living alone with an OR of 1.32 (CI 1.14–1.53). This suggests that the degree of risk was quite similar across subjective and objective indices. In contrast, social participation was associated with a decreased risk of mortality. Another prospective study suggested the risk was higher for those under the age of 65 of age (Holt-Lunstad et al., 2010).

Social isolation and loneliness may increase the risk of mortality by influencing biological and behavioral risk factors (Elovainio et al., 2017; Leigh-Hunt et al., 2017). Leigh-Hunt et al. (2017) also found a direct causal pathway for the impact of social isolation on cardiovascular disease and increased risk of post-myocardial infarction mortality. Those with the greatest social isolation presented with a two to three times higher risk of the latter compared with those with low or no social isolation. Loneliness was also a predictor for cardiovascular disease events over time in another recent study and may put vulnerable people particularly at risk (Valtorta et al., 2018). There is some evidence of association with other physical health conditions, namely cancer, low back pain and chronic obstructive pulmonary disorder, though this is less robust (Leigh-Hunt et al., 2017). Social isolation and loneliness are also associated with tobacco use, though this relationship can be unclear (Elovainio et al., 2017; Leigh-Hunt et al., 2017). Finally, social isolation and loneliness are also associated with increased risk of suicide ideation and suicide attempts, depression (including post-stroke depression) and dementia (Leigh-Hunt et al., 2017).

Overall, the evidence above suggests that these are serious social problems which the health psychology profession needs to engage with. However, these are complex problems, which rarely involve simple solutions. Thus, the first part of the dual role of the health psychology profession may be to understand the current role of civic participation in efforts to address these issues, including impacts on the health and well-being of those involved in such initiatives. The second part may involve creating interventions to help increase effective participation in such initiatives, which may involve increasing a sense of prosocial purpose in participants.

## Purpose

### Purpose as an Over-Arching Construct

Interest in purpose in the field of positive psychology has increased greatly since Damon et al. (2003) published their seminal paper on the development of purpose in adolescence. There is some variation in the definition of purpose in the literature, though there are some authors who consider a prosocial component to be key in purpose development (Damon et al., 2003). They argued that people may pursue goals and values which promote a higher purpose which can involve, for example, a sense of creativity, morality, or spirituality. Though purpose involves meaning and sense-making processes, it is goal-directed and thought to involve a stable, enduring intention toward accomplishment (Damon et al., 2003). Additionally, McKnight and Kashdan (2009) suggest purpose can act as central, life-organizing aim which stimulates goals, manages behavior, and provides meaning. They also argue that whilst it may provide a sense of direction to follow, this is not essential for living as people may choose not to follow their purpose, as they can be genetically healthy but not have any sense of this. However, if they do identify a purpose in life, they may be drawn to this, as it can be attractive and involves appetitive motivation (McKnight and Kashdan, 2009). That is, they feel a desire to engage with their perceived purpose in life and associated activities in anticipation of achieving a hoped-for positive event or possibility. This is in contrast to avoidance motivation which involves conducting actions to minimize the risk of aversive experiences such as failure, fear, sadness or worry (Elliot, 1999).

### Theoretical Basis for the Development of Purpose

Some theories suggest that purpose as a construct is a facet of meaning in life (Park, 2010). Park (2010) synthesized a model of meaning in life based on a number of previous theories about this construct. Park suggested, in her model of meaning in life, that people possess orienting systems which provide the motivation and the means to cognitively and affectively interpret salient experiences, which constitutes global meaning. These global meaning structures encompass current beliefs and affect, including a sense of justice, control, predictability, and coherence. This also incorporates global goals consisting of processes aimed toward desired outcomes or maintenance of current circumstances. However, people may face situations which stress or test their existing global meaning structures, which may lead to appraisal of situations and ascribe meaning to them using current schemas. The degree to which those appraised meanings differ from their existing global meaning structures can determine the degree of distress, or other form of emotional arousal, this can cause. This results in meaning or sense-making to reduce discrepancies and integrate these experiences into current cognitive structures, restore a sense of coherence, and to feel that life is worthwhile.

**Figure 1:**
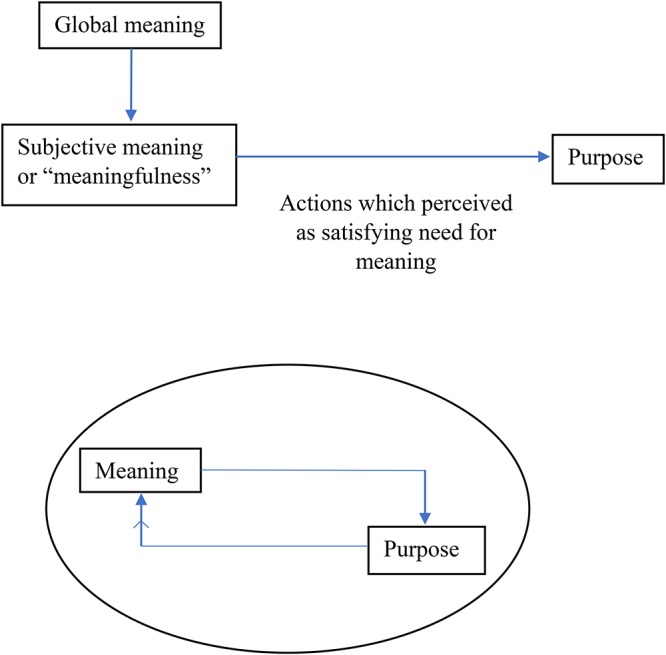
The proposed relationship between purpose and Meaning in life according to McKnight and Kashdan (2009) and Park (2010). A strong sense of meaning can lead to development of a sense of purpose. This subsequently sustains a sense of meaning through a bidirectional relationship.

Park (2010) situates purpose within “subjective meaning” as a facet of the model. More specifically, she argues that “meaningfulness” leads to a sense of purpose or direction in life, and that purpose is derived from perceiving actions to be aimed at achieving a specific goal. In contrast, McKnight and Kashdan (2009) argue that a sense of meaning is a pre-requisite for gaining a sense of purpose, but once the latter has been created or “found,” they sustain each other in a bi-directional relationship (see Figure 1 for an illustration of this proposed relationship). For the purposes of this review, we will use the latter definition as it demarcates the difference between meaning in life and purpose more clearly as distinct constructs for examination, which may aid generation of hypotheses to be tested.

Purpose may also be differentiated from goal setting as the latter has an end outcome in sight, whereas the former is thought to provide a broader motivational base and to act as an organizing principle for goal-directed behavior. McKnight and Kashdan (2009) also theorized a stronger sense of purpose may be supportive of health promoting behavior and protective against issues such as substance misuse, as these can impact goal-directed activity. They argued it may also mediate anxiety as it promotes greater exposure to stressors and can support adaptive coping strategies such as problem solving and help seeking.

### The Prosocial Element, Key to Purpose or One Facet?

Purpose is a complex, multi-faceted construct which may or may not have a prosocial element. Some researchers argue that to demonstrate purpose the accomplishments sought by individuals need to have perceived value to the self and beyond the self. Otherwise these are classed as self-oriented goals (Damon et al., 2003; Moran, 2009, 2010). However, this is not necessary for the definition adopted by McKnight and Kashdan (2009), who do not focus on morality *per se*, but social acceptability. A sense of purpose that is not socially acceptable, they argue, leads to goals that are progressively more difficult to achieve, due to the increasing severity of social sanctions. However, a purpose generally considered acceptable in wider society leads to setting goals which may receive positive social support and thus become progressively easier to achieve overall. However, Damon et al. (2003) and Moran (2009, 2010) focus on a definition of purpose which includes considerations of impact on the world ‘beyond the self.’ Their implicit focus was on prosocial purpose, but their definition also includes striving to make a wider impact through creative pursuits whose social repercussions may be largely unanticipated until their end products circulate into wider use (for example the implications of social media on connectedness and privacy).

For the purposes of this paper, to aid ease of interpretation and hypothesis testing, we will adopt the definition of purpose proposed by Hill et al. (2010). They identified four purpose orientations. The first is a “prosocial orientation,” which involves having the tendency to help others and positively influence societal structures. The second, “financial orientation,” consists of goals oriented toward financial success and material wealth accumulation. The third, a “creative orientation,” has been defined as having artistic goals and tendencies toward originality. The fourth and final facet or type, “personal recognition orientation,” has been defined as the desire for recognition and respect from peers for achievement. Hill et al. (2010), in their study of university students during their studies and at follow-up found that, firstly, prosocial and creative orientations were moderately related to each other, but there were no other significant overlaps between the different subgroups in their purpose orientations. Secondly, they found that prosocial orientation had the strongest impact on subjective well-being and generativity in mid-adulthood (participants were in their thirties at follow-up), followed by creative orientation, whereas financial or personal recognition orientations did not have any significant impact on this. Furthermore, they found that only a prosocial orientation predicted significant personal growth and integrity in later adulthood. This study was conducted with students attending a relatively wealthy college which espoused prosocial values, so further testing is needed with more representative samples. Nevertheless, there is mounting evidence that having a strong sense of purpose can have a positive impact on subjective well-being in individuals (Jenkinson et al., 2013; Czekierda et al., 2017). There is also some evidence that a strong sense of meaning and purpose may also reduce the risk of mortality in older adults, by creating a perception of having “value to others” (Cohen et al., 2016).

### Prosocial Purpose and Wider Societal Issues

It has been proposed that increasing the awareness amongst health professionals and the general population of the impact of climate change on health and well-being may encourage individuals and communities (locally and nationally) to change the way they live for the sake of self-preservation (Brittany et al., 2018). Two of the potential avenues suggested involving health promotion, for example, encouraging the uptake of diets rich in fruit and vegetables, and the creation of “greener” spaces which may entail changes in transportation and increased physical activity (Brittany et al., 2018). This may be an obvious opportunity for the health psychology profession to become involved as healthy eating and physical activity are key foci in the field (Conner and Norman, 2015). However, it is also plausible that the relationship is bidirectional; individuals and communities may adopt healthier behaviors for ethical and environmental reasons as well as to improve their health, and it may be important for health psychologists to understand this.

One of the most significant changes to health behaviors societies can currently make is to reduce or eliminate the consumption of animal or animal-based products, as this practice is a significant contributor to carbon emissions (Gerber et al., 2013), freshwater shortages, deforestation, and land degradation (Steinfield et al., 2005). Additionally, civic participation in conservation, “clean up” activities to manage plastic pollution, and reforestation may also have mitigating effects on climate change, with health implications for the individuals and communities involved, albeit this is not to suggest they will eliminate the impact of deforestation or plastic production completely (Chazdon, 2008; Cloutier et al., 2018). However, civic participation in these activities may be a significant contributor to restoring ecosystems (Chazdon, 2008) and localized efforts to reduce pollution can be effective in increasing awareness of the impact of individual actions on the environment. This can also result in potential “downstream” behavioral changes for those who become involved. An example from previous research was a reduction in littering in an area for up to 6 months after participation in a “clean up project” project’ there (Uneputty et al., 1998; Wyles et al., 2017).

There is a growing trend for consumers in Western countries to substitute meat with alternative sources of protein and adopt plant-based diets in greater numbers (Hoffman et al., 2013; Radnitz et al., 2015; Stoll-Kleemann and Schmidt, 2017; Malek et al., 2019). Cross-sectional research suggests that people may make key dietary changes for environmentally related reasons, rather than health reasons (Hoffman et al., 2013; Radnitz et al., 2015). This may be due to rapidly increasing awareness of the environmental costs of meat: 37% of the participants in an Australian sample indicated they were willing to reduce or eliminate the intake of meat or animal-based products from their diet for environmental reasons (Malek et al., 2019). However, there are several issues with this research. Firstly, these studies are cross-sectional and involve the use of convenience sampling, including online surveys, to suggest these trends are taking place. Studies by Joyce et al. (2012) and Neff et al. (2018), the latter with a more nationally representative sample in the United States, suggest that climate change is indeed becoming a more important reason for people to take up a vegan or vegetarian diet, but this still only applies to a relatively small segment of the population. Joyce et al. (2012) argued that only 3% of people in some studies were aware of the environmental impact of a meat-based diet, but 12% of the people surveyed by Neff et al. (2018) had changed their diet for environmental reasons. However, 51 and 50% had changed their diet for health and cost reasons. This is a trend that needs monitoring over time as it develops and may well indicate that there is a slow and steady increase in the proportion of the population who are changing their diet for environmental reasons.

Additionally, meat consumption may be increasing in developing countries with strongly growing middle classes (OECD, 2016). Furthermore, both Neff et al. (2018) and Malek et al. (2019) found a significant proportion of their samples had no intention of reducing their meat intake at all. This suggests that whilst there is some evidence of changes in behavior in a growing proportion of the population, there may yet be those who are “committed” meat eaters. Interestingly, Malek et al. (2019) found this group were least likely to believe that meat intake was linked to climate change. Significant barriers to reducing meat intake were enjoyment, cost, and concerns about the nutritional adequacy of a vegetarian or vegan diet (Joyce et al., 2012; Neff et al., 2018). These are all “egoistic” or self-oriented concerns, which may suggest that one approach to address the reluctance to reduce meat intake is by framing it as a form of prosocial behavior.

It is also important to understand whether prosocial purpose can be cultivated to increase engagement in initiatives to reduce social isolation and loneliness amongst vulnerable groups. A greater sense of purpose is thought to positively impact positive effect and psychological flexibility (McKnight and Kashdan, 2009; Hill et al., 2016, 2018). Prosocial purpose is thought to involve greater empathy, self-awareness, awareness of others, and recognition of the impact of decisions and behavior on others (Moran, 2009). These are important qualities in addressing social isolation and loneliness because social isolation and loneliness are associated with poorer mental health, depression and suicide ideation and attempts (Leigh-Hunt et al., 2017). There also exists a certain stigma in self-reporting loneliness, which may necessitate a more sensitive approach to addressing this issue with those experiencing it Newall and Menec (2019).

Additionally, purpose is hypothesized to be self-regulatory in nature and may lead to greater “grit,” or perseverance, toward goal-directed behavior (McKnight and Kashdan, 2009). This has been found in research with university students (Hill et al., 2018). In a similar vein, Hoffman et al. (2013) found that for those who had already adopted a vegetarian diet, undertaking this for ethical reasons was associated with greater length of vegetarianism, conviction, and maintenance of ethical motivation. This may be because this was underpinned by having a greater sense of prosocial purpose. Bronk et al. (2009) also found that purpose “beyond-the-self” (which encompasses prosocial purpose but also includes creative purpose) was associated with greater hope. Hope was defined by the presence of the feeling that one has the means to act successfully and that certain things may be worth working toward, regardless of the outcome, and can therefore be motivating. Future health psychology research may also test whether having a strong sense of prosocial purpose leads to an increased likelihood of sustaining behavior change over time, as this can be difficult to achieve and motivation may be a key factor (Alageel et al., 2018; Kwasnicka et al., 2019).

### Intervention Development to Cultivate Prosocial Purpose to Address These Issues

There are some promising avenues for intervention development to strengthen prosocial purpose. Burrow et al. (2014) found that asking participants to spend a few minutes writing about what they felt may be their purpose in life was enough to enhance this temporarily, though it is unclear if this has a lasting impact and is not specific to prosocial purpose. Bundick (2011) also conducted individual interviews with emerging adults in a university student population to assess if having a reflective discussion about their purpose in life could facilitate purpose development and found that it increased goal-directedness and life-satisfaction relative to the control group. Although it did not increase purpose identification in this group, it may be a useful inclusion in a wider intervention to cultivate prosocial purpose in individuals.

Additionally, research by Uneputty et al. (1998), Stepenuck and Green (2015), and Wyles et al. (2017) indicates it may be possible to enhance a sense of prosocial purpose by involving volunteers and citizens in beach “clean-up” activities and environmental monitoring programs. Certainly, Stepenuck and Green (2015) noted participants reported increased awareness of local environmental issues and a greater sense of purpose through participation in meaningful activities with tangible outcomes (Stepenuck and Green, 2015). Participants in the research by Wyles et al. (2017) also reported feeling a greater sense of meaning following this exercise, which may be thought of as a precursor to prosocial purpose (Damon et al., 2003; Park, 2010).

It may be that these activities increased awareness, empathy, and reflection, which stimulated meaning-making processes. Empathy may be targeted, in the context of climate change, toward vulnerable populations who are already experiencing the negative effects of climate change and who may be most deeply affected by this issue in the future despite contributing least to greenhouse gas emissions. It may also be possible to foster empathy toward non-human animals and plant life (Adams, 2014; Barrett et al., 2015; IPCC, 2018). The writing exercise undertaken by participants in Burrow et al. (2014) may have linked abstract learning with reflection, whilst Uneputty et al. (1998); Stepenuck and Green (2015), and Wyles et al. (2017) operated through the experiential learning-reflection pathway to meaning-making, according to experiential learning theory (Kolb, 1984).

However, difficulties may be encountered in recruiting people to these activities before prosocial purpose has been cultivated. To address this, it may also be the case that a simulation of some of the consequences of climate change (e.g., more extreme weather events, degraded land) or of social isolation may also increase empathy and reflection (Vanlaere et al., 2012) through experiential learning. This may become more easily accessible through technology, e.g., by creating appropriate scenarios using virtual reality.

There may also be opportunities to stimulate meaning-making and help cultivate prosocial purpose through guided discussion-based approaches (i.e., more abstract methods), such as narrative therapy (White, 2000). Narrative therapy, as conceptualized by White (2000), involves exploring the stories that people tell about themselves and their lives and co-construction of an alternative life-narrative between therapist and client. This may, amongst other elements, involve reflection on and consideration of identity and values. Prosocial purpose may also involve increased self-awareness and empathy toward others (Moran, 2009), so facilitating identification and development of these attributes may also be important for cultivating it in participants. Gucciardi et al. (2016) examined the utility of storytelling approaches for chronic disease self-management in group settings, using a “narrative-based” approach. These approaches involved generating individual reflections on experience by encouraging participants to write their own life narratives, followed by sessions involving guided group discussion of these stories to facilitate re-interpretation and shared meaning-making of their experiences. In interventions aimed at cultivating prosocial purpose, these may be useful steps to take. This may be followed by a discussion on the topic of prosocial purpose and how this may be “put in action,” as purpose is thought to be goal-oriented, whereas meaning is not (Damon et al., 2003; McKnight and Kashdan, 2009). Interestingly, Spiegel et al. (2007), studying breast cancer survivors, invited participants to reflect on how they would re-order their “life priorities” as part of their intervention. They aimed to support sense-making processes and did find that participants reported a greater sense of meaning but may have inadvertently also gained an increased sense of purpose through this activity.

The research in therapeutic contexts may also indicate that it is possible to increase prosocial purpose in vulnerable groups as well as in the general population. This may also potentially be beneficial to the participants’ physical and mental health (Hill et al., 2016, 2017) as well as helping to address wider social issues, which need to be addressed by mass participation in prosocial behavior. Moran (2009) argues that a strong sense of prosocial purpose may be a form of “intrapersonal giftedness” (at least amongst American youth) in a society that promotes self-oriented goal achievement. However, it is possible that this is not necessarily the case if more people are supported to explore and cultivate a sense of prosocial purpose to help address the social challenges we face today.

### Those Who Have Identified Their Purpose May Still Need Guidance on How to Appropriately Achieve Their Goals in Fulfilling Prosocial Purpose

Addressing climate change, social isolation and loneliness, and other important social causes, is a complex challenge. Thus, individuals who have identified their purpose may still benefit from support in understanding and deciding how they can play a role in addressing key issues. The relationship between prosocial purpose and Bandura’s concept of self-efficacy (Bandura, 1986, 2001) needs to be better understood, but previous research in the area indicates a strong sense of purpose may not necessarily lead to improved mastery. McKnight and Kashdan (2009), for example, argue that purpose can act as an organizing principle for goal setting, but that being thwarted in achieving a perceived purpose can be distressing. Bronk et al. (2009) also suggest individuals may need support and guidance on how to achieve their purpose as this support may strengthen hope in the process of finding and cultivating purpose. In contrast, self-efficacy is enhanced through increasing mastery and achievement (Bandura, 1986) in individuals or through vicarious learning. That is the focus of the latter construct, whereas individuals may have a strong sense of prosocial purpose in addressing issues because of their importance, even if it appears unlikely or impossible that they will achieve their desired end goals (Bronk et al., 2009).

Firstly, research is unclear on whether an underlying ethical motivation for dietary change necessarily leads to healthier eating. Both Ogden et al. (2007) and Radnitz et al. (2015) have found that high ethical motivation for dietary change was associated with greater dietary restriction (i.e., complete avoidance of certain food items rather than restricting calorie intake). However, Radnitz et al. (2015) also found that those who were motivated to adopt a vegan diet for ethical reasons were more likely to eat soy, foods rich in vitamin D, and fluids high in phytochemicals, which were classed as healthy options by the researchers. Conversely, those motivated by health reasons ate more fruit, drank more fruit juice, and ate fewer sweets, though fruit also has high natural sugar content. There was no difference in vegetable intake between both groups. These findings suggest that overall diet quality was similar in both groups. In contrast, Ogden et al. (2007) found that being ethically motivated was not significantly associated with successful overall dietary change in university students, apart from the avoidance of certain food groups altogether. Only the latter was significantly associated with positive attitudes to change. Overall, the evidence tentatively suggests that those taking up vegan or vegetarian diets to address climate change may still need support and guidance to follow a healthy diet.

The benefits of a healthy majority plant-based diet should not be underestimated. A recent systematic review by Toumpanakis et al. (2018) of randomized controlled trials investigating the impact of plant based dietary interventions (at least 90% plant-based food intake) on type 2 diabetes management underscores this. They found those in the intervention groups experienced greater decreases in HbA1c levels (i.e., greater blood sugar control) relative to control groups. There was also a statistically significant decrease in medication and insulin use for those on a plant-based diet, but no changes in medication for control groups. Participants from the intervention group also reported an increased quality of life, a greater decrease in neuropathy, greater increases in self-esteem, nutritional efficacy and general efficacy; they considered that adopting a plant-based diet was an acceptable lifestyle change. This suggests that increasing prosocial purpose and supporting healthy dietary change can be mutually beneficial for individuals and wider society. It also suggests that interventions to increase prosocial purpose can, and should, include vulnerable groups and those with chronic illnesses in their efforts.

Secondly, Newall and Menec (2019) also argued that though social isolation and loneliness are distinct concepts, those who experience social isolation and loneliness are particularly vulnerable and potentially hard to reach. Although they may be highly motivated to engage in programs aiming to reduce loneliness and secure better-quality relationships, they can often be hidden due to the few opportunities they have in engaging with others. By contrast, theoretically, those reporting high social isolation but low isolation may have greater choice in the matter and be potentially less motivated to join help programs, or not report loneliness at all due to stigma. Those who experience isolation may be prone to more severe health difficulties due to their social disconnection, so perhaps public health campaigns or social marketing campaigns may be helpful, to raise general awareness of the health value of larger networks or having contacts in case they have a fall. This may also “prime” development of goal-directed impetus for prosocial purpose development in individuals as this is a key facet of the construct (Damon et al., 2003; McKnight and Kashdan, 2009).

Finally, those who are lonely, despite having large social networks, may have experienced relationship loss or loss in general, toxic relationships or unrealistic expectations regarding their social contacts, and would benefit from psychological intervention to address these issues. Regardless of the group they belong to, those who experience these issues may need help to address their root causes. For example, some may have poor access to wider communities (whether due to transport, income or poor physical or mental health). Thus, individuals with a high level of prosocial purpose may require opportunities and support to learn about the issues involved and develop skills to help address social isolation and loneliness in an appropriate manner. Having a strong sense of purpose toward a cause which one feels one cannot act to address can cause significant distress (McKnight and Kashdan, 2009). Conversely, trying to guide people to address too many social issues, particularly in quick succession, may be self-defeating as they may not have the resources to address them all (McKnight and Kashdan, 2009). Interventions aiming to cultivate prosocial purpose need to be mindful of this. Researchers and practitioner psychologists may also need to monitor the impact of this overload on the well-being of the individuals involved who have developed a sense of prosocial purpose. Early evidence indicates that purpose may foster positive affect in students (Hill et al., 2016) and is also associated with lower impact of stressors on subjective well-being and reduced negative affect amongst middle-aged adults (Hill et al., 2017). However, purpose may not be a panacea for well-being (Burrow et al., 2018) and these findings need to be replicated when examining the impact of prosocial purpose on the physical and mental health of adults.

Cultivating pro-social purpose by fostering engagement with environmental initiatives such as monitoring changes in local biodiversity, “clean ups” to reduce inappropriate plastic waste disposal, or gardening to support the creation of “green” spaces may also merit exploration (Uneputty et al., 1998; Stepenuck and Green, 2015; Wyles et al., 2017; Cloutier et al., 2018). These activities can have a positive impact on local environments and may also help to reduce climate change and land degradation; and improve air quality, urban and rural spaces, and habitats for wildlife, potentially creating carbon sinks (Uneputty et al., 1998; Stepenuck and Green, 2015; Wyles et al., 2017; Cloutier et al., 2018). Participating in environmental volunteering may also lead to an increase in participants’ physical activity levels, in contrast to other types of volunteering (Jenkinson et al., 2013), so may have additional physical health benefits. This may also facilitate the political impetus to lobby for the creation of healthier environments, such as pedestrianized roads, with further “downstream” benefits for local communities, and so may be mutually beneficial to individuals and wider society (Cloutier et al., 2018). This may be important, as changes to individual behaviors alone will not be enough to address these environmental issues: wide-ranging social changes are needed to mitigate climate change and its effects (Adams, 2014; IPCC, 2018). However, both Stepenuck and Green (2015) and Cloutier et al. (2018) noted the presence of expertise to guide these processes. Health psychologists may benefit from closer cross-collaboration with psychologists in other disciplines, environmental scientists, and local conservation groups or “greening initiatives” to support the uptake of environmental volunteering amongst individuals and communities they work with more effectively.

### Prosocial Purpose and Self-Efficacy

Both prosocial purpose (Damon et al., 2003) and self-efficacy (Bandura, 1986, 2001) are thought to be self-regulatory processes. They can also direct goal setting behavior and self-monitoring of progress. However, as noted to in the section above, a person may have a strong sense of prosocial purpose without perceived knowledge or understanding of how to achieve this (McKnight and Kashdan, 2009). They may maintain their efforts toward this due to its perceived importance (Bronk et al., 2009), but lacking mastery and achievement of the knowledge and skills to achieve their purpose-related goals may contribute to distress associated with not fulfilling that purpose (McKnight and Kashdan, 2009). In contrast, the focus of self-efficacy is that of increasing mastery in the development of knowledge and skills in a particular area through practice and vicarious learning (Bandura, 1986). McKnight and Kashdan (2009) hypothesized that having a strong sense of purpose may decrease risk of behaviors which may impede or disrupt progress toward achieving purpose-oriented goals. This may be through facilitating earlier identification of an issue which may impact achievement of those goals, earlier help-seeking and through reducing the attractiveness of those alternatives (McKnight and Kashdan, 2009). There is some emerging research to support this view, for example, indicating that stronger purpose is associated with a reduced risk of having eating disorders and, for those who are diagnosed with such, reduced symptom severity (Marco et al., 2017). It is possible that high general self-efficacy may also have a protective effect through a shared cognitive pathway with purpose through self-regulation and organizing goal-directed behavior, so these may be moderately correlated in research. This may then reduce the risk of behaviors which impede mastery development and enhance those which may aid goal achievement, including health behaviors, as has often been found in the literature (Conner and Norman, 2015). Alternatively, it may be that individuals with a stronger sense of purpose have a greater propensity to increase their self-efficacy due to their enhanced self-regulatory capabilities, which may lead to the acquisition of these benefits for their well-being, with self-efficacy acting as a mediating variable.

### Prosocial Purpose and Collective Well-Being

Roy et al. (2018) suggested that both meaning in life and purpose contribute to collective well-being through stimulating contribution to community life, through activities such as volunteering and civic engagement. However, this may be an over-simplification. Prosocial purpose may be key to improving collective well-being, when knowledge and skill mastery has been achieved. To get to this point, it may be important not only for individuals to have increased self-efficacy (Bandura, 1986), but also for communities to have greater collective efficacy (Bandura, 2001). Individual self-efficacy is theorized to be an important contributor to collective efficacy, but the latter also additionally involves the sense of being able to work together effectively as a group or community to achieve shared goals and outcomes (Bandura, 2001). Thus, collective efficacy may mediate the relationship between increased prosocial purpose and collective well-being. It is also possible that collective efficacy and collective well-being may then have a bi-directional relationship, so they mutually sustain each other. This may also be an important consideration for the development of community health psychology interventions to foster prosocial purpose in the general population.

### A Note on Morality: Prosocial Purpose Versus “the Greater Good”

It is important not to conflate cultivating prosocial purpose with working toward ‘the greater good’ in this context. This is mainly because whilst there is a focus beyond the self and an aim to help others in both concepts, prosocial purpose does not involve deliberately harming a smaller group of individuals to serve a greater cause. This includes perpetrating physical harm and violence due to negative morality. It can lead to loss of life and has been implicated in many past atrocities such as those committed by the Nazis during World War II. Thus, it is important when aiming to cultivate prosocial purpose to set appropriate boundaries and expectations regarding acceptable behavior to achieve good causes, e.g., by following the Hippocratic oath of “First, do no harm.” Fostering compassion and belonging, rather than “othering” certain groups, may also be important in this context, by supporting empathy, whether that is through simulation or facilitating personal contact with others, particularly those that face marginalization such as people in poor health (Mariano, 2014).

### Hypothesis Testing

Based on these findings regarding prosocial purpose in the research literature and prior key theorizations on how sense of purpose may be developed, a number of hypotheses will now be put forward for testing. These relate to cognition and behaviors of individuals with a strong sense of prosocial purpose in relation to wider social issues. This will be followed by a proposal of a framework to guide intervention development to foster prosocial purpose in individuals.

**Figure 2:**
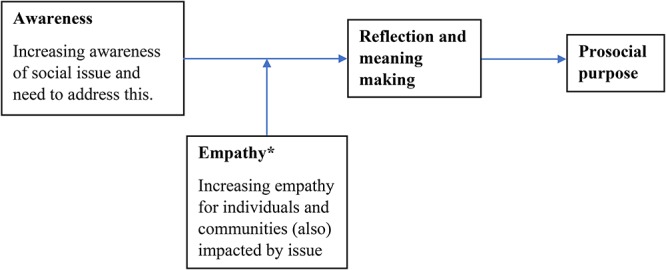
Proposed model for intervention development. ^∗^Previous research by Vanlaere et al. (2012) suggests experiential learning can be particularly effective in engendering empathy.

• People with a stronger sense of prosocial purpose are more likely to change their diet for ethical reasons, including because they aim to address climate change.
• Individuals in the general population with stronger prosocial purpose will also experience greater subjective well-being and generativity relative to people with other purpose orientations over time, replicating findings with a graduate sample (Hill et al., 2016).
• The sense of prosocial purpose is stable over time in individuals and can lead to greater perseverance with actions to mitigate climate change and efforts to reduce social isolation and loneliness, building on Damon et al. (2003), McKnight and Kashdan (2009), and Hill et al. (2018).
• Prosocial purpose is moderately correlated with self-efficacy in individuals, as they share some self-regulatory processes and both involve goal-directed activity (Bandura, 1986; Damon et al., 2003). However, prosocial purpose differs from self-efficacy in that it involves perseverance with goal-directed activity aimed at achieving a given purpose at times when positive impact may be perceived as unlikely or impossible to achieve due to the relationship between prosocial purpose relationship and hope (Bronk et al., 2009).
• Greater prosocial purpose contributes to collective well-being (Roy et al., 2018), but other purpose orientations (creative, achievement or financial), as defined by Hill et al. (2010), do not. Indeed, financial purpose may actually decrease collective well-being in respect to climate change by promoting participation in activities which contribute to environmental degradation.
• Greater presence of prosocial purpose in individuals is important for creating collective well-being through the mediating effect of collective efficacy, elaborating on the proposed relationship between purpose and collective well-being suggested by Roy et al. (2018).
• Prosocial purpose contributes to collective well-being through “downstream” impact on civic engagement in initiatives to reduce threats facing wider society, including climate change, and reducing social isolation and loneliness.
• It may be possible to cultivate prosocial purpose in individuals by activating meaning or “sense-making” processes as this is a pre-requisite for purpose development. To activate meaning-making processes it is important to stimulate reflection on discrepancies between the desired state of the world and the actual state of affairs in a way that enhances personal relevance and empathy, following Park (2010); please see Figure 2 for an illustration of this process.

• Empathy and reflective processes may potentially be activated in several ways. Firstly, by guided group discussion and support to explore discrepancies between the social challenges humanity face and how participants would like the world to be. Secondly, empathy and reflection may be stimulated by experiential learning regarding the issues we face (Kolb, 1984) including by simulation of lived experience of for example, social isolation (Vanlaere et al., 2012). Thirdly, they may learn experientially by participating in prosocial activities (Uneputty et al., 1998; Wyles et al., 2017). This may be followed by discussion on finding a sense of purpose through meaningful action to achieve change, as purpose is goal-directed, whereas meaning is not (Damon et al., 2003; Bronk et al., 2009; McKnight and Kashdan, 2009).
• It is possible to foster prosocial purpose in vulnerable populations with chronic physical and mental health issues as well as the general population. “Intrapersonal giftedness,” which involves having enhanced empathic and reflective capacities (Moran, 2009), is not a prerequisite in participants for this to occur.
• Interventions to foster prosocial purpose will result in improved subjective physical and mental well-being, including positive affect and psychological flexibility, of participants.
• Individuals who develop a stronger sense of prosocial purpose may have more creativity in solution finding to achieve their purpose, as prosocial and creative purpose orientations are related (Hill et al., 2010).
• Individuals may still need guidance to support their self-efficacy in conducting prosocial behavior when they develop a stronger sense of purpose due to the complexity of the issues they are engaging with. Not being able to achieve the purpose one is following can cause distress (McKnight and Kashdan, 2009).

## Conclusion

Possessing a strong sense of prosocial purpose may be important for individuals to engage in addressing wider social issues including climate change and social isolation and loneliness. These are two very important areas for the health psychology profession to become involved in addressing due to their wide-ranging health implications. Prosocial purpose may increase subjective well-being over time, but environmental volunteering in particular may also have additional physical health benefits. Greater prosocial purpose may also help explain why there may exist a slow but steady increase in the numbers of people in Western countries who are reducing their intake of meat and dairy products. However, changes in dietary habits need to be monitored over time and prosocial purpose needs to be established as an antecedent to such changes through longitudinal research exploring the relationship between prosocial purpose, dietary change, and diet quality. Prosocial purpose may also lead to increased perseverance with behavior change in individuals and it may also relate to increased self-efficacy through shared self-regulatory processes, making it a potentially effective target for intervention development. Those with greater prosocial purpose may also need support and guidance on how they can help address wider social issues such as climate change and social isolation and loneliness. These issues are not solely the responsibility of individuals to address but require concerted efforts in individual, group, and community contexts. Supporting the development of prosocial purpose may also contribute to increased collective efficacy and collective well-being in communities, so they are better equipped to manage these issues. Greater prosocial purpose may also empower groups to participate in non-violent political and social activism, which is also important as wider social institutions also need to change their practices to address these issues effectively. This is especially so in relation to climate change, which requires wide-ranging changes in our societies if we are to have any success in mitigating its negative effects and restraining temperature rises so that they are manageable for humans and the wider natural environment.

## Author Contributions

KB responsible for conceptualization of the review, conducting literature searches, writing the article, and applying for funding to publish the article. TT editing and revising content and making significant intellectual contributions, supervising the submission, and providing approval for publication. Both authors agreed to be accountable for the content of the work.

## Conflict of Interest Statement

The authors declare that the research was conducted in the absence of any commercial or financial relationships that could be construed as a potential conflict of interest.
